# Implementation of an Evidence-Based Exercise Program for Older Adults in South Florida

**DOI:** 10.1155/2016/9630241

**Published:** 2016-10-05

**Authors:** Richard C. Palmer, Anamica Batra, Chelsie Anderson, Timothy Page, Edgar Vieira, Laura Seff

**Affiliations:** ^1^Robert Stempel College of Public Health and Social Work, 11200 SW 8th Street, Miami, FL 33199, USA; ^2^College of Nursing and Health Sciences, 11200 SW 8th Street, Miami, FL 33199, USA

## Abstract

*Introduction*. This study aimed to examine how well an evidence-based physical activity program could be translated for wide scale dissemination and adoption to increase physical activity among community-dwelling older adults.* Methods*. Between October 2009 and December 2012, reach, fidelity, dosage, ease of implementation, and barriers to translation of EnhanceFitness (EF) were assessed. To assess effectiveness, a pretest-posttest design was used to measure increases in functional fitness (chair stands, arm curls, and the up-and-go test).* Results*. Fourteen community-based agencies offered 126 EF classes in 83 different locations and reached 4,490 older adults. Most participants were female (72%). Thirty-eight percent of participants did not complete the initial 16-week EF program. The 25% who received the recommended dose experienced an increase in upper and lower body strength and mobility. Further, participants reported high satisfaction with the program.* Conclusion*. EF was successfully implemented in a variety of settings throughout South Florida and reached a large number of older adults. However, challenges were encountered in ensuring that those who participated received a program dose that would lead to beneficial gains in functional fitness.

## 1. Introduction

Regular physical activity can promote physical and psychological wellbeing and reduce risk of disability and vulnerability to chronic diseases in older adults [1–5]. Nevertheless, adults over 50 years of age are the least physically active segment of the US adult population [6–8]. Evidence-based exercise programs designed for older adults show promise as a way to increase opportunities for safe and effective physical activity [9]. However, little is known about how well these programs, which were tested and evaluated using controlled trials, are translated for wider use in community settings [10]. It is important to know if research-based programs will maintain their effectiveness when they are implemented in community settings [11]. Further, accurate interpretation of outcomes depends on knowing which aspects of the intervention were delivered and how faithful the delivery was to original program design [12].

The Healthy Aging Regional Collaborative (HARC) was established by the Health Foundation of South Florida (HFSF) to increase the adoption of evidence-based programs that promote and preserve the health of older adults in South Florida. Among the programs selected was EnhanceFitness (EF), which is an evidence-based group exercise program for older adults of all fitness levels [13, 14]. EF aims to help participants improve overall functional fitness and personal wellbeing. Intervention studies have documented the efficacy of EF and have shown that EF improves performance on fitness assessments, perceived physical functioning, social functioning, mental health, and decreases bodily pain [15–18]. Limited evidence exists demonstrating that EF can be translated to community-based settings and still achieve improvements in fitness and wellbeing [19]. Therefore, we investigated the ability of HARC to implement and sustain the EF program in South Florida over a three-year period. We examine if HARC was able to retain program fidelity, recommended dosage and exposure to the program, and comparable gains in functional fitness.

## 2. Methods

### 2.1. Setting

The Healthy Aging Regional Collaborative was created to increase local community capacity to offer accessible health promotion programs to older adults in South Florida. HARC was established by the HFSF and was designed to (1) disseminate evidence-based health promotion programs to address inactivity and chronic health conditions in older adults in South Florida; (2) establish a local group of trained professionals and lay leaders with the capacity to provide a range of evidence-based health promotion programs to older adults on an ongoing basis; and (3) provide a foundation for planning, coordination, technical assistance, and peer learning for present and future collaborative members. Community-based agencies that were interested in participating in HARC had to apply to HFSF and received funding to offer evidence-based programs, including EF. Funding covered licensing for use of the selected evidence-based program, instructor training, program implementation materials and equipment, and support for the administrative costs of implementation, including procurement of sites, instructors and participants, data collection, and data entry. Funded agencies were responsible for delivering EF. Agencies identified suitable sites to offer EF classes (i.e., churches, senior housing, senior centers, community centers, and parks) as well as recruited program participants.

### 2.2. EnhanceFitness Overview and Implementation

A one-hour EF class includes a 5-minute warm-up, a 20-minute low impact aerobic workout, a 5-minute cooldown, a 20-minute strength training workout, and 10 minutes of stretching and is offered three times per week. Instructors who were certified by a nationally recognized fitness organization (i.e., YMCA, ACE, or ACSM) and who received training in EF procedures at a two-day training conducted by EF program developers and EF master trainers led classes. All instructors were required to teach EF as outlined in EF training materials provided by the program developer. Individuals over the age of 50 who were interested in participating in EF were enrolled by agencies as long as they were cognitively and physically able to participate in the EF program.

### 2.3. Data Collection and Management

Multiple sources of data were collected to evaluate how well HARC agencies were able to implement EF. Descriptive/demographic data form, a health history, a fitness check (FC), and program satisfaction survey were collected. Participant data were collected at enrollment, with a second FC and the evaluation scheduled to be completed 16 weeks after enrollment as prescribed by the EF program. Program managers and instructors collected all participant data. All staff who administered the forms and collected data were trained regarding the meaning and importance of the items and shown the correct way to administer the three functional tests included on the FC during EF training sessions. Program managers submitted class registrations, new and ongoing participant data, and participant attendance records to HARC's evaluation team. An annual survey of program managers was also conducted online. Program fidelity was monitored by trained observers who observed randomly selected classes and then completed a fidelity observation checklist aimed at identifying implementation issues.

Prior to receiving any data from agencies, HARC's evaluation team received approval from the Institutional Review Board at Florida International University to conduct the evaluation of HARC.

### 2.4. Measures

The number of agencies, sites, classes, and participants were tracked as measures of EF implementation. Demographic characteristics of participants were self-identified on a form that included questions regarding gender, age, race and ethnicity, highest level of education, annual personal income, living situation (alone or with others), marital status, and primary spoken language. Participant satisfaction was measured using the satisfaction survey developed and provided by EF.

Effectiveness of the EF program was assessed based on changes in measures of functional fitness (30-second arm curl to assess upper extremity strength, the 30-second chair stand to measure lower extremity strength, and the timed eight-foot up-and-go circuit to measure balance and mobility), self-reported general health, and self-reported frequency (days per week) of exercise of at least 30-minute duration.

Additional measures included (1) the BRFSS general health measure, with a response scale from 1 = poor to 5 = excellent [20]; (2) self-reported number of days of exercise of at least 30-minute duration in a week, including EF sessions; and (3) a single item on the second FC to measure the self-reported degree of improvement in physical abilities (walking, bending, strength, moving around, and doing the activities you want to do) as a result of EF program participation, reported on a 10-point scale where 1 is no improvement and 10 is great improvement. Attendance records provided data regarding dose, average number of sessions attended by participants, and average duration of participant enrollment.

The annual survey of HARC program coordinators served as a process evaluation tool and identified implementation issues that agencies in HARC experienced. Questions specific to implementing EF were included each year and only agencies who offered the EF program completed these items. Items assessed ease of implementing the EF program, challenges with implementation, assessed participant recruitment activities, and also assessed long-term adoption and sustainability.

Observations of 30% of randomly selected classes assessed fidelity to the program design. Observers were staff members of the evaluation team and were trained on program specifications through a detailed review of the instructor manual. As part of the training, observers would accompany an experienced observer and interrater reliability would be assessed. Observers used a structured checklist that was based on the instructor manual and was developed by the evaluation team, EF master trainers, and HARC member agencies. The checklist specified observable elements related to the class environment, instructor, duration, and site. Each element was marked as observed or not observed. Discrepancies from the prescribed exercise routine were noted in a comment section.

### 2.5. Data Analysis

**Figure 1:**
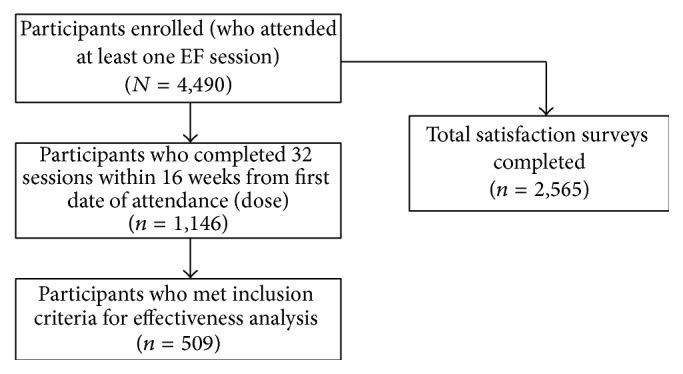
Number of participants by inclusion criteria.

Of the 4,490 enrolled participants, 1,146 received the dose of the intervention proposed by Belza and colleagues [13] (Figure 1). Exclusion criteria for the current study include (1) no data for the pretest, the posttest, or both; (2) a first FC that did not fall within 30 days before and seven days after the first attendance date (to assure that baseline data reflected actual abilities prior to any benefits received from attending EF sessions); (3) a second FC completed before the recommended dose was attained; and (4) more than six months between the first and second fitness checks. With these exclusions, the sample used to calculate participant outcomes included 509 participants (Figure 1).

Data were extracted from the online database and imported into SPSS 22.0 for analysis. Data was first assessed for normality and outliers. Data on participant satisfaction, self-reported improvement in physical abilities, participant characteristics, participant attendance metrics, and program manager survey and fidelity observation data were analyzed using descriptive statistics. To test specific hypotheses about program effectiveness, the general linear model (GLM) was chosen to assess within-subject changes in outcome measures (arm curls, chair stands, and 8-foot up-and-go) at baseline and 6 weeks and controlled for potential differences in performance site [21].

## 3. Results

A total of 4,490 older adults enrolled and attended at least one EF session between October 1, 2009, and December 31, 2012. The majority of enrolled participants were women. Participant ages ranged from 29 to 107, with a mean age of 74.8 years (SD ± 9.9). The racial/ethnic composition of participants was diverse, with non-Hispanic whites and Hispanics making up the two largest groups (Table 1). The number of agencies, classes, sites, and participants, tracked in each of the first three years of the HARC as measures of EF implementation, are shown in Table 2, by year. Total number of participants reached increased every year. The largest number of new participants was reached in Year 1. After an expected reduction of new participants in Year 2, the number of new participants increased in Year 3, but not to the Year 1 level. At the end of the three-year period, nine agencies were funded and active, offering 70 ongoing EF classes at 56 sites (Table 2).

### 3.1. Fidelity

**Figure 2:**
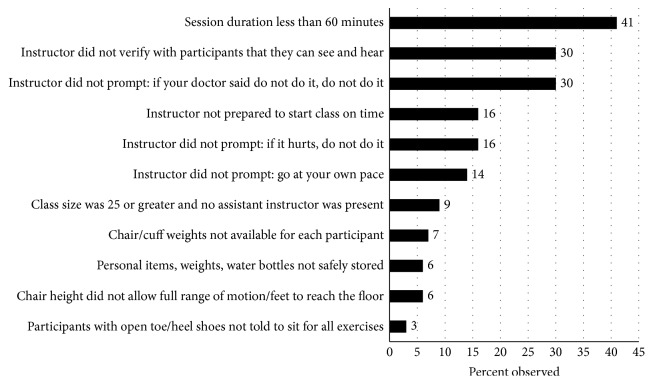
Frequency of fidelity concerns identified.

Sixty-nine EF classes were observed for fidelity by the evaluation team between, accounting for nearly 33% of all EF classes offered during the time period. Almost half of the observed classes, 47.8%, had no significant fidelity concerns in the measured dimensions. The most frequently cited concerns related to instructors not using key phrases developed to ensure participant safety, including checking if participants could see and hear the instructor (30%), and reminding participants not to do exercises their physician advised against (30%), not to do any exercises that were painful (16%), and to exercise at a pace that was comfortable (14%). Additionally, 41% of observed sessions lasted less than 60 minutes (Figure 2).

### 3.2. Dose

Of 4,490 participants enrolled in the HARC EF program between October 1, 2009, and December 31, 2012, who attended at least one session, an average of 26% received the minimum recommended dose [13] (Table 2). The ratio of completers to new participants enrolled declined in each of the three years. Other measures, potentially relevant in terms of dose, were examined. Three percent of enrolled participants attended only one session and 38% stopped attending within the initial 16 weeks. The average time between enrollment and last date of attendance was 1.1 years. The average number of sessions attended per participant was 59.

### 3.3. Ease of Implementation and Barriers

In the Year 1 program manager survey, six of seven program managers responsible for implementing EF reported that implementation was difficult or very difficult on a five-point scale from very easy to very difficult. In the Year 2 and Year 3 surveys, more than half of the program managers indicated that implementation was moderate, that is, half-way between very easy and very difficult on the measurement scale, showing that ease of implementation improved over time. Implementation barriers most frequently cited over the three years remained relatively constant and included funding, inadequate staff, inadequate client interest, cultural norms of the community, and difficulty in recruiting class instructors. The most frequent barriers identified for recruiting participants included difficulty maintaining consistent participation and the seasonal residence of some participants in the South Florida area. The most frequent barriers to instructor recruitment included availability for the three weekly one-hour sessions, finding individuals who were committed to the EF program, and lack of transportation.

### 3.4. Effectiveness

Analysis revealed that participants who completed the recommended dose and received a posttest after participating in the program for four months significantly improved their scores on the three physical ability measures, self-reported general health, and frequency of daily exercise of at least 30-minute duration after participating in EF program (Table 3).

### 3.5. Participant Satisfaction

When asked on the second FC about improvements in physical abilities as a result of their EF participation, 71.5% of participants indicated improvement. Among the 2,565 EF participants who completed a program evaluation, 89% were satisfied or highly satisfied with the program. Additionally, 97% responded that they would take the class again and 92% would recommend the class to a friend.

## 4. Discussion

The HARC initiative described here is the largest scale translation of EF to a broad cross section of community agencies and settings within the same geographical area to date in the United States. Data reported here suggest that HARC was able to successfully implement EF throughout South Florida and reach a large number of older adults over the three-year period. These findings indicate that this effort to form a collaborative, sharing resources, training, and knowledge, was an effective approach for translating an evidence-based program to a wide array of community settings and sites.

Unlike controlled trials of EF, data reported here are based on the real world implementation of EF in a variety of community settings throughout South Florida. It was important to investigate if the community-based EF implementation produced participant changes similar to those reported in controlled EF efficacy studies. Our findings are consistent with prior controlled studies of EF and other fitness programs [13, 15, 16]. Participants in this study demonstrated statistically significant improvements in upper body strength, lower body strength, functional mobility, self-reported health status, and frequency of daily exercise of at least 30-minute duration over the follow-up period.

As with any adoption of a health promotion program, the potential for changes and deviations in the curriculum is a concern. Adaptation is a normal process, yet it can cause a program to be less effective if changes are made that undermine the program's effectiveness. In our evaluation, we assessed fidelity to determine how accurately EF agencies and instructors implemented the program. Surprisingly, very few concerns were identified for the core elements of the program. The majority of observed concerns involved safety warnings that instructors are supposed to use throughout the class.

Our evaluation did identify several areas that warrant comment and further investigation. A participant recruitment challenge relating to male participants existed. Only about 15% of participants were male, while the percentage of males in the total population in the three targeted Florida counties is much higher, at nearly 44% [22]. This rate of male participation is similar to findings of other programs that serve older adults, although there is limited knowledge regarding why older men do not participate in health promotion programs [23, 24]. There is a need for research that explores how to overcome male resistance to such participation. Additionally, of the process issues identified, perhaps the most significant were frequency of attendance and attrition. There appears to be a problem with participants achieving the recommended dosage (32) sessions within a 16-week period that has been associated with functional fitness and mobility benefits [13]. Almost 41% of all EF participants in this region stopped attending within the initial 16-week period. Moreover, for those who did continue through the initial 16 weeks and beyond, only 26% attended at least 32 sessions in the first 16 weeks. In both cases, participants did not receive the dose that has been suggested as being optimal to obtain all the benefits associated with exercise and strength training. For EF to be effective, participants must participate regularly to achieve maximum benefits. Subsequently, more research is warranted to better understand the elements that affect attendance patterns of older adults at ongoing exercise programs and to explore strategies that effectively address these attendance barriers.

Although findings show that EF can be effectively implemented by a collaborative in South Florida and that participants can achieve benefits consistent with clinical trials if they participate regularly, this evaluation has certain limitations. As this was a community-based implementation and not a controlled research study, a randomized design could not be used. Participants self-selected into the program, so they may not be representative of the larger older adult population in South Florida. Bias could also be an issue if functional test results reflect the impact of health-promoting activities that participants engaged in other than EF. A large number of participants did not have documented follow-up measures, which reduced data available for analysis of fitness test results. Finally, functional test data are potentially subject to measurement bias because different instructors collected data on outcome measures. However, the rigorous training on data collection tools prior to being certified to teach an EF class, use of the same tool, and ongoing fidelity monitoring of instructors reduces the chances of measurement bias.

Even with these limitations, this study had strengths. The program was implemented by multiple agencies, and the diverse racial/ethnic mix of the South Florida participants is likely different than demographic profiles found in previously described evaluations. Many measures of implementation used indicated successful introduction of the programs and ongoing fidelity to program design. A longer-term retrospective study of sustained fidelity and the program's impact on participant physical fitness, health status, frequency of exercise, and healthcare costs is needed to confirm that outcomes reported here endure over longer time periods.

## 5. Conclusions

Health promotion efforts targeting older adults are essential for improvement in quality of life, functional ability, and healthcare cost savings. HARC's innovative approach to rapid EF implementation on a wide scale shows that EF can be introduced in many different settings and delivered successfully. Moreover, there is reason to believe that community-based EF programs have the potential to offer access to an evidence-based physical activity with proven health benefits to a large percentage of older adults in the community. However, efforts are needed to ensure that community-based agencies that offer EF reinforce the importance of continued attendance to ensure the most benefit to program participants and that implementers continue to assess implementation fidelity of the program to the original design tested in controlled trials.

## Figures and Tables

**Table 1:** Characteristics of EnhanceFitness participants.

	*N* = 4,490^a^
Gender	
Female	3,216
Male	862
Age	
<60 years	176
60–69 years	948
70–79 years	1,462
80–89 years	936
90 years and over	276
County of residence	
Broward	1,588
Miami-Dade	1,920
Monroe	645
Race-ethnicity	
Hispanic/Latino	677
Haitian/other non-Hispanic Caribbeans	144
White, non-Hispanic	794
Black, non-Haitian	188
Other	44
Marital status	
Married/partnered	1,625
Single/not partnered	2,343
Annual income	
<$15,000	1,089
$15,000–$24,999	450
$25,000–$49,999	404
$50,000–$75,000	185
>$75,000	151
Education	
Less than high school	613
High school graduate	919
Some college	1,031
College graduate or higher	1,404
Other variables	
Frail/disabled	470
Has Medicare	2,641
Has Medicaid	807

^a^Reported values may not add up to total participant *N* due to missing data.

**Table 2:** HARC EnhanceFitness process measures.

Measure	Year 1 *N*	Year 2 *N*	Year 3 *N*	All years *N*
Participants	1,528	1,961	2,127	4,490
Percent completers	29.4%	24.3%	21.8%	25.5%
Agencies offering EF classes	8	9	9	14
EF classes offered	59	73	79	126
Classes observed for fidelity	18	25	26	69
Sites where EF classes were offered	51	60	63	83

EF: EnhanceFitness.

**Table 3:** EnhanceFitness effectiveness measures.

Fitness test	*N* ^a^	Baseline mean (SD)	Follow-up mean (SD)	Change	*P* value
Number of chair stands in 30 seconds	491	11.78 (4.09)	15.41 (5.36)	3.63	<.001
Number of arm curls in 30 seconds	499	15.92 (5.85)	20.76 (7.34)	4.84	<.001
Number of seconds to complete up-and-go	487	8.62 (4.69)	7.40 (4.28)	−1.22	<.001

SD: standard deviation; N/A: not applicable.

^a^Reported values do not add up to total participant (*N* = 509) due to missing data.
